# Development of potent reversible selective inhibitors of butyrylcholinesterase as fluorescent probes

**DOI:** 10.1080/14756366.2019.1710502

**Published:** 2020-01-08

**Authors:** Stane Pajk, Damijan Knez, Urban Košak, Maja Zorović, Xavier Brazzolotto, Nicolas Coquelle, Florian Nachon, Jacques-Philippe Colletier, Marko Živin, Jure Stojan, Stanislav Gobec

**Affiliations:** a Faculty of Pharmacy, University of Ljubljana, Ljubljana, Slovenia; b Faculty of Medicine, Institute of Pathological Physiology, University of Ljubljana, Ljubljana, Slovenia; c Département de Toxicologie et Risques Chimiques, Institut de Recherche Biomédicale des Armées, Brétigny sur Orge, France; d Institut Laue Langvein, Grenoble, France; e CNRS, CEA, IBS, Université Grenoble Alpes, Grenoble, France; f Faculty of Medicine, Institute of Biochemistry, University of Ljubljana, Ljubljana, Slovenia

**Keywords:** Butyrylcholinesterase, inhibitor, probe, fluorescence

## Abstract

Brain butyrylcholinesterase (BChE) is an attractive target for drugs designed for the treatment of Alzheimer’s disease (AD) in its advanced stages. It also potentially represents a biomarker for progression of this disease. Based on the crystal structure of previously described highly potent, reversible, and selective BChE inhibitors, we have developed the fluorescent probes that are selective towards human BChE. The most promising probes also maintain their inhibition of BChE in the low nanomolar range with high selectivity over acetylcholinesterase. Kinetic studies of probes reveal a reversible mixed inhibition mechanism, with binding of these fluorescent probes to both the free and acylated enzyme. Probes show environment-sensitive emission, and additionally, one of them also shows significant enhancement of fluorescence intensity upon binding to the active site of BChE. Finally, the crystal structures of probes in complex with human BChE are reported, which offer an excellent base for further development of this library of compounds.

## Introduction

Alzheimer’s disease (AD) is the most common form of senile dementia, and its prevalence is expected to increase further in the coming decades due to ageing of the populations in the Western world^1^. AD is characterised by progressive impairment of cognition that is accompanied by behavioural decline, which results in loss of the ability to perform basic activities of daily life^2^. As such, it causes distress for patients and caregivers, and it is becoming a large economic burden on society^2^.

The pathogenesis of AD is complex. It includes accumulation of the amyloid β protein (Aβ) and abnormal modifications and accumulation of the hyperphosphorylated protein tau, which is accompanied by oxidative stress. This leads to synaptic loss, selective neuronal death, and decreased brain concentrations of specific neurotransmitters, most notably of acetylcholine (ACh)^3^. Following diagnosis of AD, it generally leads to death within 3 to 9 years; however the initial silent and asymptomatic stages start some 20 years before the onset of symptoms^4^. Currently, the available treatments for AD include cholinesterase inhibitors and memantine, an N-methyl-D-aspartate (NMDA) receptor inhibitor. Unfortunately, these medications do not change the course of the disease nor the rate of patient decline, despite improving the quality of life for both patient and caregiver^5^.

Cholinergic synapses are particularly affected by Aβ neurotoxicity, and the synaptic loss induces decreased brain ACh concentrations, which correlate in time with the cognitive impairment that is characteristic of AD. ACh is the neurotransmitter at cholinergic synapses, where its activity can be terminated by two cholinesterases: acetylcholinesterase (AChE) and butyrylcholinesterase (BChE). The activity of BChE is increased in certain regions of the brain of patients upon AD progression^6^. On the other hand, the expression of AChE decreases throughout the brain along with disease progression, thus making BChE an attractive target for the development of selective inhibitors for the treatment of AD, particularly in the later stages. Additionally, fewer side effects are expected for BChE inhibitors compared to the current AChE inhibitors due to the absence of BChE at cholinergic synapses outside of the brain; namely, at neuromuscular junctions and in the parasympathetic autonomous nervous system^6^. Furthermore, as BChE accumulates in Aβ plaques, it has the potential to serve as a biomarker of AD^6^. The Aβ plaques are a central hallmark of AD, although a significant number of elderly people, and even young people, can have Aβ plaques without cognitive impairment^7^.

Future development of better treatments and more rapid and accurate diagnostic tools, and ultimately the prevention of AD, depends on a full understanding of the pathophysiology of AD, especially during the period before the onset of symptoms. The cholinesterases have important roles in the pathogenesis of AD, although their exact roles and their interplay in relation to the other hallmarks of AD remain poorly understood. New molecular tools to study cholinesterases might provide new insights into the pathophysiology of AD. Fluorescent probes are particularly useful here, due to the high sensitivity of fluorescence techniques and the potential for visualisation through various fluorescence microscopy techniques. Numerous AChE-specific fluorescent inhibitors have been reported, some of which are irreversible and some of which are competitive^8–11^. A tacrine-based fluorescent inhibitor with high affinity towards both AChE and BChE has been described, and this was shown to stain Aβ plaques when used as a probe for histochemical staining of brain tissue^12^. Besides probes developed from inhibitors, two recently described BChE probes were developed as substrates for BChE^13^^,^^14^. BChE converts the nonfluorescent substrate to highly fluorescent products. Furthermore, both probes are poor substrates for AChE or other types of esterases, thus providing high selectivity towards BChE^13^^,^^14^. However, no BChE-selective probes derived from inhibitors were reported to date.

In the present study, we report on the two BChE-specific fluorescent probes that were derived from previously described highly potent and reversible BChE-selective inhibitors^15–17^. Although the incorporation of the fluorophore moieties had a negative impact on their BChE inhibition potency, the best three of these fluorescent inhibitors retained excellent activities in the low nanomolar range: probes **2A**, **3A**, and **3B**. Moreover, significantly increased fluorescent intensity was observed upon binding of probe **2A** to BChE. The crystal structures of these three best probes in complex with human BChE (huBChE) confirm the binding mode that was anticipated in the design phase.

## Material and methods

### Synthesis

Detailed schemes, synthetic procedures, and characterisation of each probe are given in the Supporting Information.

### Inhibition of huAChE, mAChE, and huBChE

The inhibitory potencies of these probes against cholinesterases (i.e. huAChE, murine AChE [mAChE] and huBChE) were determined using the method of Ellman^18^, as previously described^19^. The assays were carried out at room temperature in 100 mM phosphate buffer, pH 8.0, containing 333 µM Ellman reagent (5,5′-dithiobis(2-nitrobenzoic acid); DTNB), 500 µM substrate (huAChE/mAChE: acetylthiocholine; huBChE: butyrylthiocholine iodide [BSCh]) and huAChE/mAChE or huBChE (50 pM, 1 nM, respectively). The reactions were started by addition of the substrate, and the final dimethylsulphoxide (DMSO) carrier concentration did not exceed 1% (v/v). Formation of the yellow product was followed for 1 min as the change in the absorbance at 412 nm, using a microplate reader (Synerg H4; BioTek Instruments, Inc., USA). For screening for inhibitory activities, the compounds were assayed at 100 µM, in triplicate. The initial velocities in the absence (*v_0_*) and presence (*v_i_*) of the test compounds were calculated. The inhibitory potencies are expressed as the residual activities (RAs), according to Equation (1):

(1) $$\text{RA}\;\left(\%\right)\;=\;(v_{i}\;-\;b)/(v_{0}\;-\;b))\;\times \;100,$$

where *b* is the blank value.

For the IC_50_ measurements, serial dilutions were prepared for the compounds with RAs <50% at 100 µM. The IC_50_ values were calculated by plotting the RAs against the compound concentration according to a four-parameter logistic function, using the GraphPad Prism 8 software (GraphPad Software, USA). All of the measurements were repeated twice, each of which was in triplicate.

### Kinetic studies

#### Chemicals

Purified huBChE was a generous gift from Drs. O. Lockridge and L. Schopfer. DTNB and BSCh were from Sigma. All other reagents used were of analytical grade. The experiments were carried out at 25 °C in 25 mM phosphate buffer, pH 7.0.

#### Inhibitory effects of 2C, 3A, and 3B on butyrylthiocholine iodide hydrolysis by purified huBChE

Three compounds (**2C**, **3A**, **3B**) were tested by measuring the progress curves for BSCh hydrolysis by huBChE in the absence and presence of each of these compounds at five to seven concentrations. Assays were carried out over 10 min, in a 0.6-ml cuvette, using the method of Ellman et al^18^. The compound concentrations used were: **2C**, 0–1 µM; **3A**, 0–200 µM; and **3B**, 0–50 nM.

The concentration of purified huBChE, which was always the final addition to these assays, was ∼0.5 nM. The hydrolysis of 50 µM BSCh was followed until completion (in most cases), in the presence of 1 mM DTNB. Reaching the plateau was important to get a precise estimate of the actual added BSCh concentration. For the calculation, the molar absorption coefficient of 13800 mol^−1 ^cm^−1^ was used. All of the measurements were performed using a conventional UV/Vis spectrophotometer (Lambda45; Perkin-Elmer). Due to the experimental manipulation, a dead time of 10 s was added to each measurement, and all progress curves were extrapolated accordingly prior to the analysis.

#### Theoretical basis of the kinetics analysis

**Figure 1.**
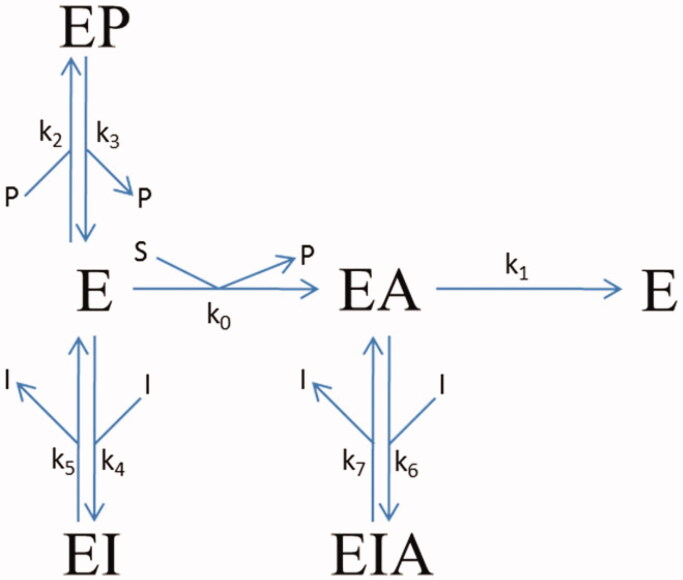
Reaction scheme for the inhibition of BSCh hydrolysis by purified huBChE in the presence of DTNB by compounds **2C**, **3A**, and **3B**. E, free enzyme; EA, acylated intermediate; S, substrate BSCh; P, all of the stoichiometrically released products (SCh-TNB, TNB^–^); and I, compounds (inhibitors) **2C**, **3A**, and **3B**. The symbols for the constants are: *k_cat_* (*k_1_*), catalytic constant for BSCh turnover; *K_m_* (*k_0_*), Michaelis constant; *K_p_ = k_3_/k_2_*, inhibition constant for binding of the product thiocholine-TNB; *K_1_ = k_5_/k_4_* and *K_2_ = k_7_/k_6_*, dissociation constants for binding of the compounds to the free and acylated enzyme, respectively.

The progress-curve analysis was performed using the ENZO web application, implemented at www.enzo.cmm.ki.si^20–22^. This programme is designed to generate differential equations from drawn reaction schemes and subsequently to fit the coefficients of these equations according to least squares methodology to reproduce the experimental data using a numerical integration algorithm. For determination of the kinetics mechanisms and parameters, the van Slyke-Cullen single intermediate reaction scheme^23^ for substrate hydrolysis by huBChE was combined with a reversible mixed inhibition mechanism (Figure 1). In all of the evaluations, the *k_1_* of 814 s^−1^, the substrate specificity constant *k_1_/K_m_* of 1.0 × 10^8^ M^−1^ ^−1^s^–t^, and the inhibition constant of 54 µM for the binding of the thiocholine-TNB (k_3_/k_2_) in the hydrolysis of BSCh by purified huBChE were constrained, as these values had been determined previously^24^.

### X-ray crystallography of huBChE in complex with 2C, 3A, and 3B

#### Crystallization

Recombinant huBChE was produced in eukaryotic cells and insect cells as described previously^25^^,^^26^. The protein was purified by BChE-specific affinity chromatography (Hupresin; Chemforase, Rouen, France), followed by size exclusion chromatography (Superdex 200; GE Healthcare), as previously described^26^. Compounds **2C**, **3A**, and **3B** were solubilised in 100% DMSO at 0.1 M, and the protein complexes were obtained by co-crystallization at 1 mM final ligand concentration (**2C**, **3B**: 0.1 M MES, pH 6.5, 2.15 M (NH_4_)_2_SO_4_, 1% DMSO; **3A**: 0.2 M ammonium acetate, 12% PEG 4000, pH 7.4) using the hanging drop method at 293 K. For **2C** and **3B**, the crystals were cryo-protected in a solution of 0.1 M MES, pH 6.5, 2.15 M (NH_4_)_2_SO_4_, 20% glycerol, 1 mM ligand, 1% DMSO before flash cooling in liquid nitrogen. For **3A**, cryo-protection was achieved with the mother-liquor complemented with 17% glycerol and 1 mM **3A**.

#### Structure determination

The X-ray diffraction data for **2C** and **3B** were collected on the ID23-1 beamline of the European Synchrotron Radiation Facility (Grenoble, France) at 100 K, with a Pilatus 6 M detector, and for **3A**, on the ID23-2 beamline at 100 K with a Pilatus 2 M detector. For **2A** and **3A**, the detector frames were processed manually with XDS^27^, and for **3B**, automatically with autoPROC^28^. Model refinement was performed using the Phoenix software suite^29^. The initial models were obtained by molecular replacement using Phaser-MR and the huBChE structure (PDB entry 1P0I), devoid of any ligands, glycans or water molecules. The electron densities in the active site gorge were examined, and allowed to undergo partial fitting with each compound. For **2C** and **3B**, ligand geometry restraints were processed using Phoenix eLBOW^30^ and the semi-empirical quantum mechanical method (AM1). For **3A**, the geometry restraints were generated using the PRODRG server^31^. Each model was refined by iterative cycles of Phoenix.refine and model building using *Coot*^32^. The huBChE structures in complex with **2C**, **3A**, and **3B** have been deposited in the Protein Data Bank under accession numbers 6R6V, 6RUA, and 6R6W, respectively.

## Results and discussion

### Structure-activity relationships

Fluorescent probes that undergo emission changes upon binding to an enzyme active site can be used as “hot ligands”; i.e., in fluorescence-based competition binding assays^33^. The environment of the binding site is generally more stable and hydrophobic than the bulk, which can result in increased intensity of the fluorescence emission and a blue-shift of the emission maximum^34^. For maximal sensitivity, a fluorophore should bind strongly and deeply into the active site. In the case of BChE, the active site is buried at the bottom of a deep aromatic gorge, at the entrance of which there is a peripheral binding site (main contributors, Asp70, Ala277). The active site features a choline-binding pocket (main contributor, Trp82) and an acyl binding pocket (main contributors, Trp231, Leu286, Phe329, Phe398), which accommodate the choline and butyrate moieties, respectively, of the substrate butyrylcholine (or its analogue, butyryl thiocholine). The catalytic activity features residues Ser198, His438, and Glu325.

**Figure 2.**
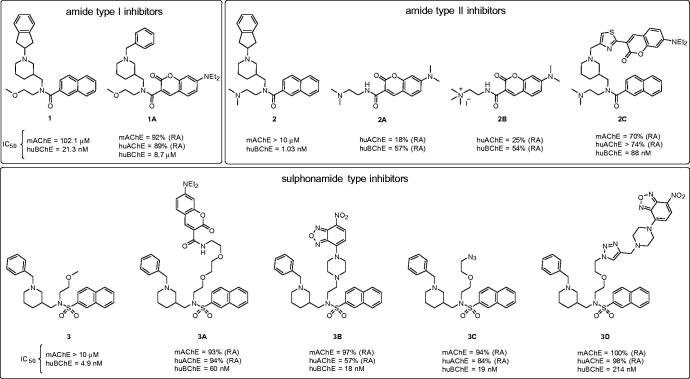
Structures of the parent non-fluorescent BChE inhibitors (compounds **1**, **2**, **3**) and the synthesised fluorescent probes (**1A**, **2A–C**, **3A–D**). The IC_50_ values and residual activities (RAs) for inhibition of mAChE, huAChE, and huBChE are given.

The design of the initial fluorescent probe based on an inhibitor of BChE (Figure 2, hit **1**) began before its crystal structure in complex with huBChE was resolved^13^. In the first attempt, the naphthalene moiety of this amide-type inhibitor **1** was substituted with 7-(diethylamino)coumarin, to yield **1A** (Figure 2). 7-(Diethylamino)coumarin was selected because of its solvatochromic properties^35^^,^^36^. Additionally, to shorten the synthetic route, the indene moiety on the inhibitor **1** was substituted with a benzyl group. In retrospect, both of these modifications were far from optimal. The crystal structure of amide-type inhibitor **1** in complex with huBChE later showed that the naphthalene was tightly bound within the acyl binding pocket, and that substitution of naphthalene with a larger moiety was bound to, and accordingly resulted in, decreased activity^15^. Replacement of the indene moiety by a benzene will also not have positively impacted on the affinity, as this eliminated the strong contribution to the binding of the cation-π interactions it established with Tyr332^13^.

With the discovery of the picomolar selective BChE inhibitor **2**^17^, the design of an inhibitor with a fluorophore part positioned in the acyl binding pocket was revisited. We hypothesised that the loss of binding affinity that resulted from the substitution of naphthalene with coumarin might be compensated for by the strong cation-π interactions between its dimethylamino group and Trp82, as seen in the crystal structure of inhibitor **2** in complex with BChE^17^. Compounds **2A** and **2B** were synthesised without the piperidine moiety of the parent inhibitor **2** (Figure 2). Additionally, the diethylamino group at position 7 of the coumarin scaffold was substituted with a less bulky dimethylamino group. To increase the interactions at the choline binding site, a compound with a permanent charge (**2B**) was designed, on the basis that compounds with a permanent ammonium cation can produce significantly stronger cation-π interactions compared with compounds with a tertiary amine moiety in the same position^37^. Indeed, the trimethylammonium fragment mimics the quarternary amine in substrate BSCh. Unfortunately, compounds **2A** and **2B** showed very low and unselective inhibition of BChE. Naphthalene was seen to be the optimal fit for the acyl binding pocket, and substitution with coumarin resulted in loss of inhibitory activity.

For the design of probe **2C** (Figure 2), a more conventional approach was therefore adopted, based on the crystal structure of inhibitor **2** in complex with huBChE. As the naphthalene and 2-(dimethylamino)ethyl moieties form crucial interactions with the active site, the indene group was replaced by a 7-(diethylamino)-3-(thiazol-2-yl)-coumarin moiety. Indeed, the indene only has non-specific interacts with the protein scaffold, and it is furthermore oriented out of the aromatic gorge, towards the bulk, which suggests more permissivity in terms of fluorescent group substitutions. Unsurprisingly, compound **2C** showed lower huBChE potency when compared to parent inhibitor **2**, which is known to be one of the best reversible inhibitors of huBChE. However, compound **2C** maintained good affinity, with an IC_50_ of 88 nM.

The binding mode of inhibitor **3** is somewhat different compared to that of inhibitors **1** and **2**^15–17^. The common property of all of these three types of inhibitors is the position of the naphthalene moiety, which fits into the acyl binding pocket of huBChE. However, the piperidine groups of inhibitors **1** and **2** points towards the entrance of the aromatic active-site gorge, while for inhibitor **3**, it fits into the choline-binding pocket and it is instead the methoxyethyl group that is oriented into the gorge. Therefore, elongation of the methoxymethyl group was the most straightforward approach to graft a fluorophore onto inhibitor **3**, to yield **3A** (Figure 2). Compound **3A** had its methoxyethyl group substituted with a triethylene glycol spacer, with 7-(diethylamino)coumarin at the distal end. This compound retained good BChE inhibitory activity, with an IC_50_ of 60 nM, but instead showed poor solubility in aqueous buffers. To avoid non-specific interactions, a more hydrophilic 1–(2-hydroxyethyl)piperazine spacer was introduced, with an environment-sensitive 7-nitro-2,1,3-benzoxadiazol-4-yl (NBD) fluorophore attached to the piperazine nitrogen. The resulting **3B** (Figure 2) had the best *in-vitro* inhibitory activity of this series, with an IC_50_ of 18 nM, and showed improved solubility in aqueous solutions compared to **3A**.

To save time in future studies by enabling easier substitution of the fluorophore moiety, we introduced a short linker with an azide into compound **3**, which can react with any fluorophore that is functionalised by an alkyne group, according to “click chemistry” (copper(I)-catalyzed alkyne-azide cycloaddition)^38^^,^^39^. Compound **3C** (Figure 2) had a short diethylene glycol linker with an azide, and it retained good inhibitory activity against huBChE. However, the introduction of a test fluorophore for compound **3D** (Figure 2) resulted in substantial loss of inhibitory activity, making this approach less attractive here.

### Kinetic studies of probes 2C, 3A, and 3B

#### Inhibition of BSCh hydrolysis by purified huBChE with 2C, 3A, and 3B

**Figure 3.**
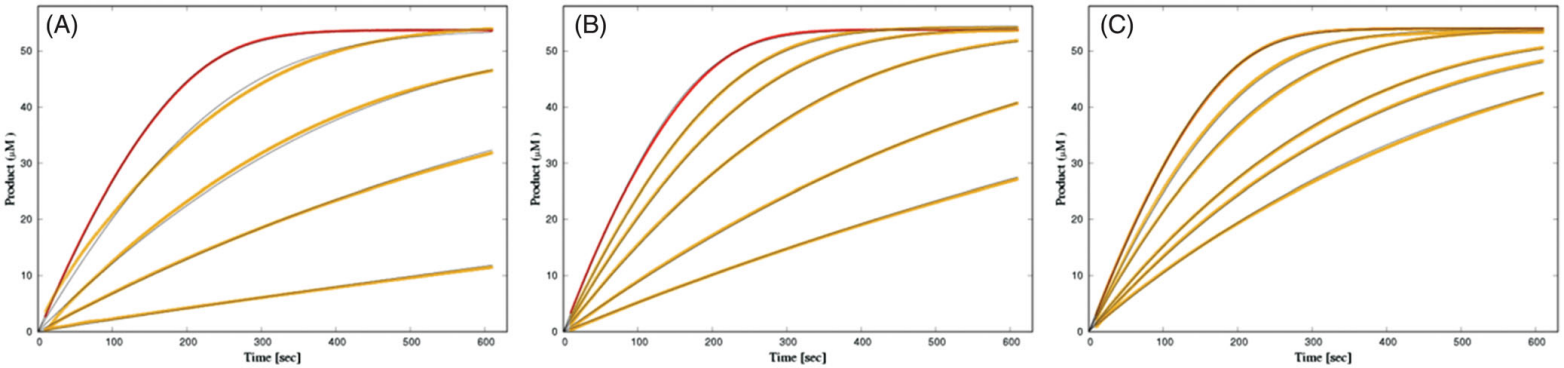
Time courses of product formation in the reactions between BSCh (54 μM) and purified huBChE (**A**, 0.47 nM; **B**, 0.49 nM; **C**, 0.43 nM) in the absence (red curves) and presence of **2C** (5, 20, 50, 200 nM) (A), **3A** (50, 100, 200, 500 nM, 1 μM) (B), and **3B** (5, 10, 25, 33, 50 nM) (C).

The actions of different concentrations of these three fluorescent inhibitors on huBChE were tested by measuring progress curves of product formation at approximately 50 µM BSCh (Figure 3). The analysis of these progress curves revealed good agreement between the experimental curves and a theoretical model that defined a competitive reaction mechanism with binding of the inhibitor to both the free and the acylated enzyme. The dissociation constants are given in Table 1, and they show significantly lower affinities of all of the ligands for binding to the acylated enzyme (i.e. *K_2_*).

**Table 1.** Characteristic dissociation and rate constants for inhibition of huBChE by the fluorescent probes according to reaction scheme in Figure 1.

Probe	*K_1_* (nM)	*K_2_* (nM)
**2C**	3.27 ± 0.04	42.7 ± 1.5
**3A**	63.0 ± 0.5	296 ± 2
**3B**	4.63 ± 0.02	96.6 ± 2.0

### Crystal structure of huBChE in complex with probes 2C, 3A, and 3B

**Figure 4.**
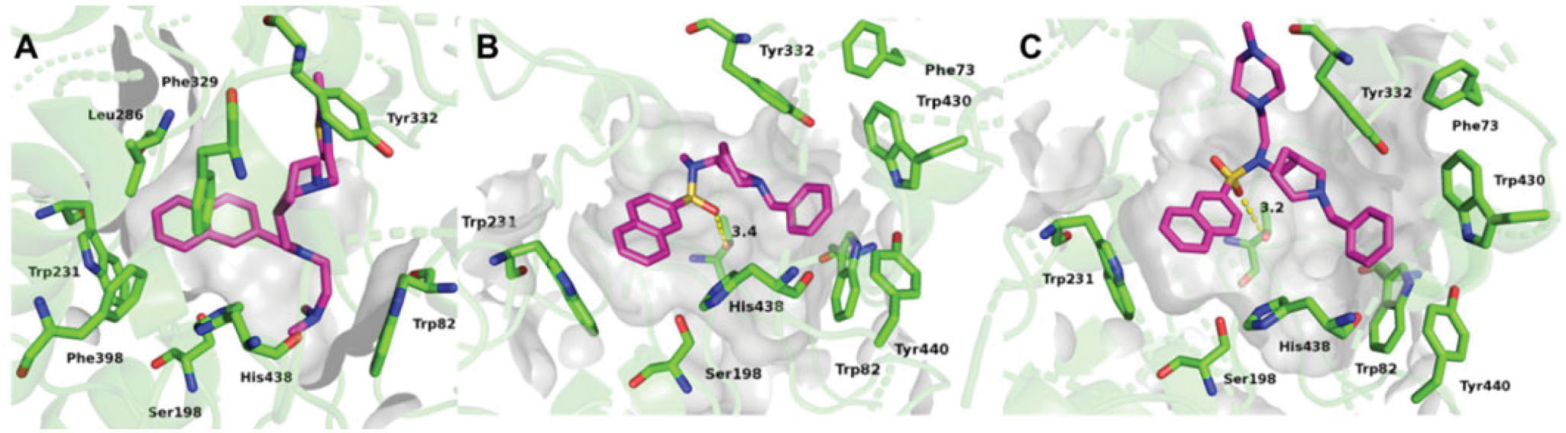
Crystal structures of **2C** (A), **3A** (B), and **3B** (C) (purple stick models) bound to huBChE (green ribbon model; PDB codes 6R6V, 6RUA, 6R6W, respectively). Key residues in the active site are shown as green sticks. (B, C) The polar H-bond between Thr120 and the sulphonamide moiety of compounds **3A** and **3B**, respectively, is shown as yellow dashes (distance: B, 3.4 Å; C, 3.2 Å).

**Figure 5.**
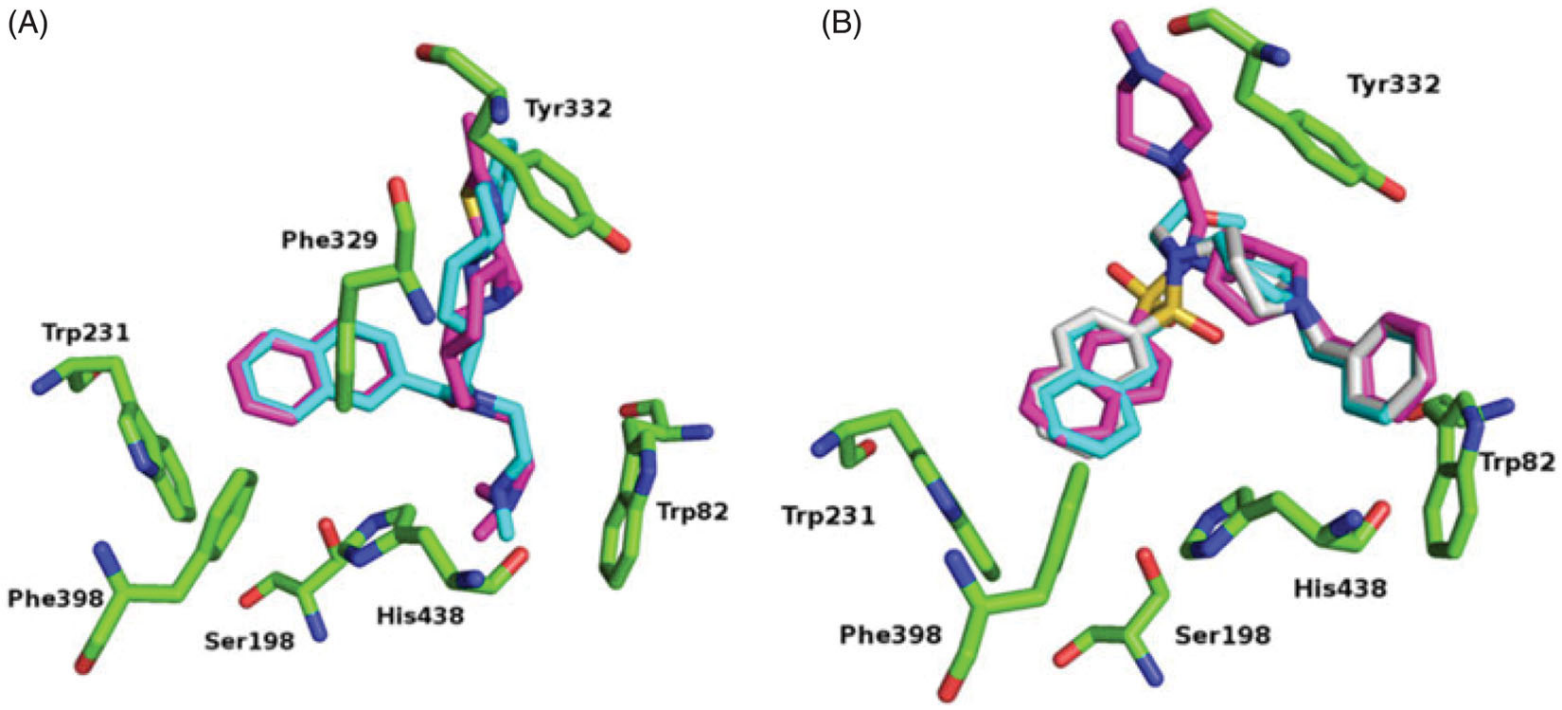
Alignments of crystal structures, with key residues in the active site, shown as green sticks. (A) Compound **2C** (purple stick model; PDB code 6R6V) and parent naphthalene inhibitor **2** (cyan stick model; PDB code 5NN0) in their complexes with huBChE. (B) Compounds **3A** (gray stick model; PDB code 6RUA), **3B** (purple stick model; PDB code 6R6W) and parent sulphonamide inhibitor **3** (cyan stick model; PDB code 5DYW) in their complexes with huBChE.

When compared to the previously determined structure of huBChE in complex with parent inhibitor **2** (PDB code 5NN0)^17^, the fluorescent naphthalene–carboxamide derivative **2C** adopts a similar binding mode, whereby the naphthalene moiety is positioned in the acyl-binding pocket through a T-stacking–like interaction with Trp231 (Figure 4(A)). The piperidine moiety interacts slightly deeper in the active-site gorge, stacking between Phe329 and Tyr332 (Figure 5(A)), while the dimethylamine moiety engages in cation-π interactions with Trp82. The remainder of the molecule is less defined. While the thiazole ring is positioned close to the parental indene moiety, no clear electron density is seen for the fluorescent coumarin-derivative moiety, which indicates that it does not interact with the protein scaffold and is likely to be free to adopt a variety of conformations, all of which will be characterised by the coumarin exposed to the bulk. The naphthalene–sulphonamide derivatives **3A** and **3B** have similar binding modes, where the naphthalene moiety points towards the acyl-binding pocket (Figures 4(B), 5(B)). The piperidine moiety is inverted compared to the naphthalene carboxamide of **2C** (5NN0), although it retains the same orientation as in the parent sulphonamide (5DYW)^16^ (Figure 5(B)), where the benzyl ring protrudes into a highly hydrophobic pocket that is formed by residues Phe73, Trp82, Tyr332, Trp430, and Tyr440. As seen for the complex structure of parent inhibitor **3** with BChE, the sulphonamide oxygens of **3A** and **3B** form H-bonds with the hydroxyl group of Thr120. The piperazine ring of **3B** points out of the aromatic gorge, towards the bulk; hence, as in the complex structure of BChE with **2C**, no clear electron density is seen for the presumably highly flexible fluorescent NBD moiety. Similarly, no clear electron density is seen for the fluorescent coumarin and the triethyleneglycol linker of **3A**, and only the first carbon attached to the sulphonamide nitrogen was modelled.

### Characterisation of fluorescence properties

**Figure 6.**
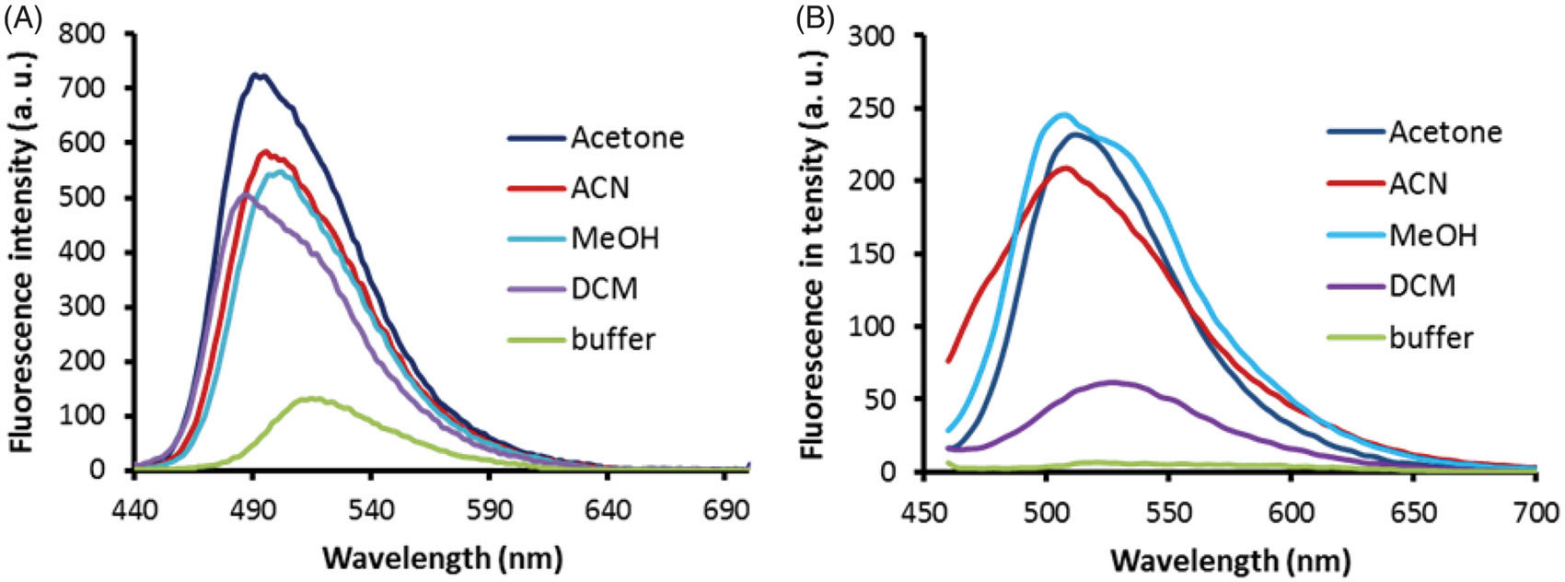
Fluorescence spectra of probes **2C** (A) and **3B** (B) at 1 μM in the various solvents (as indicated). Excitation wavelengths: 440 nm, 380 nm, for probes **2C**, 3**B**, respectively. a.u., arbitrary units.

Only probes **2C** and **3B** had both good inhibitory potencies and sufficient aqueous solubility, and so these were further evaluated for their fluorescence properties. The low solubility of probe **3A** might indeed lead to non-specific interactions with huBChE and limit is use, and it was therefore not included further. On the basis that the active site appears to be more hydrophobic than the bulk, the optimal probe should show strong emission in lipophilic solvents and no emission in polar solvents. In this respect, probe **3B** proved ideal, as it showed strong emission in all of the lipophilic organic solvents, except dichloromethane, and very low fluorescence intensity in phosphate buffer at pH 8 (Figure 6(B)). Similarly, the emission intensities of probe **2C** were also dependent on the solvent, although to a lesser extent than for probe **3B**. However, probe **2C** also showed substantial emission in phosphate buffer (Figure 6(A)).

**Figure 7.**
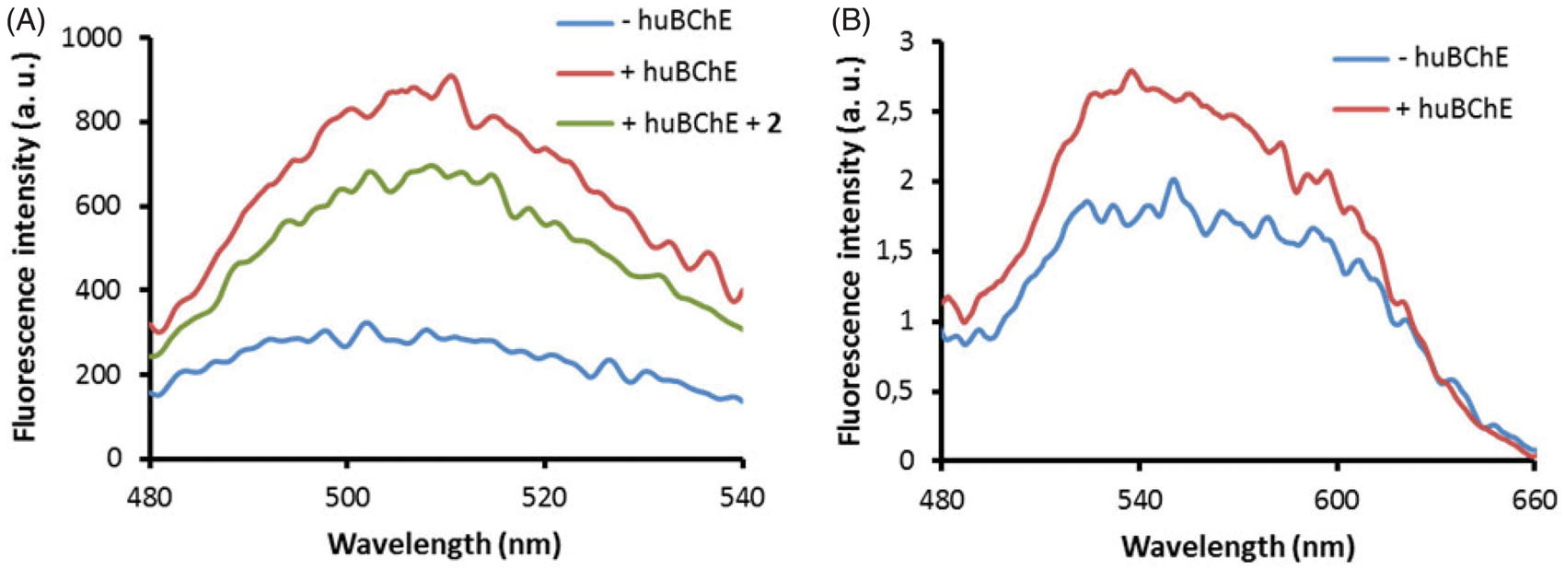
Fluorescence spectra of probes **2C** (A) and **3B** (B) at 1 μM in buffer without huBChE (blue), with huBChE at 500 nM (red) and with huBChE at 500 nM and inhibitor **2** at 1 μM (green). a.u. – arbitrary units.

The fluorescence properties of probes **2C** and **3B** were then examined when they were bound to huBChE. Probe **2C** showed relatively strong emission in the buffer, and the addition of huBChE induced an approximately two-fold enhancement of its fluorescence intensity (Figure 7(A)). This mirrored the differences in the emission of probe **2C** between the buffer solution and the organic solvents (Figure 6(B)). Improvements to the ratio of the fluorescence intensities between the bound and unbound probe might be obtained by the introduction of a more environment-sensitive fluorophore that can interact with the protein scaffold, in place of the 7-(diethylamino)-3-(thiazol-2-yl)-coumarin here, which does not. Addition of inhibitor **2** dislodged probe **2C** form the active site and decreased the emission of **2C**, nonetheless, the emission of **2C** was still substantially higher compared to the emission of probe **2C** in buffer. Most probably this is due to unspecific binding to protein surface and titration study (see Supplemental material) confirms this. Future development of probe **2C** should thus be orientated towards lowering the lipophilicity, which should, in turn, reduce unspecific binding.

The weak fluorescence emission of probe **3B** in the buffer was only marginally increased upon binding to huBChE, which indicated that the NBD fluorophore did not undergo significant changes in polarity. The crystal structure of probe **3B** with huBChE confirmed this, as no full electron density was recorded for the NBD fluorophore and the linker of probe **3B**. The NBD fluorophore would appear to lie in the polar environment at the entrance of the active site gorge, and surrounded by aqueous medium. For future development of probe **3B**, it will be necessary to position the NBD fluorophore closer to the central scaffold, and to graft in additional functional groups that will position the fluorophore in a more hydrophobic environment. This will establish further interactions with the protein scaffold, thus providing the potential for a more pronounced increase in fluorescence emission upon binding to huBChE.

### Rat brain slices

**Figure 8.**
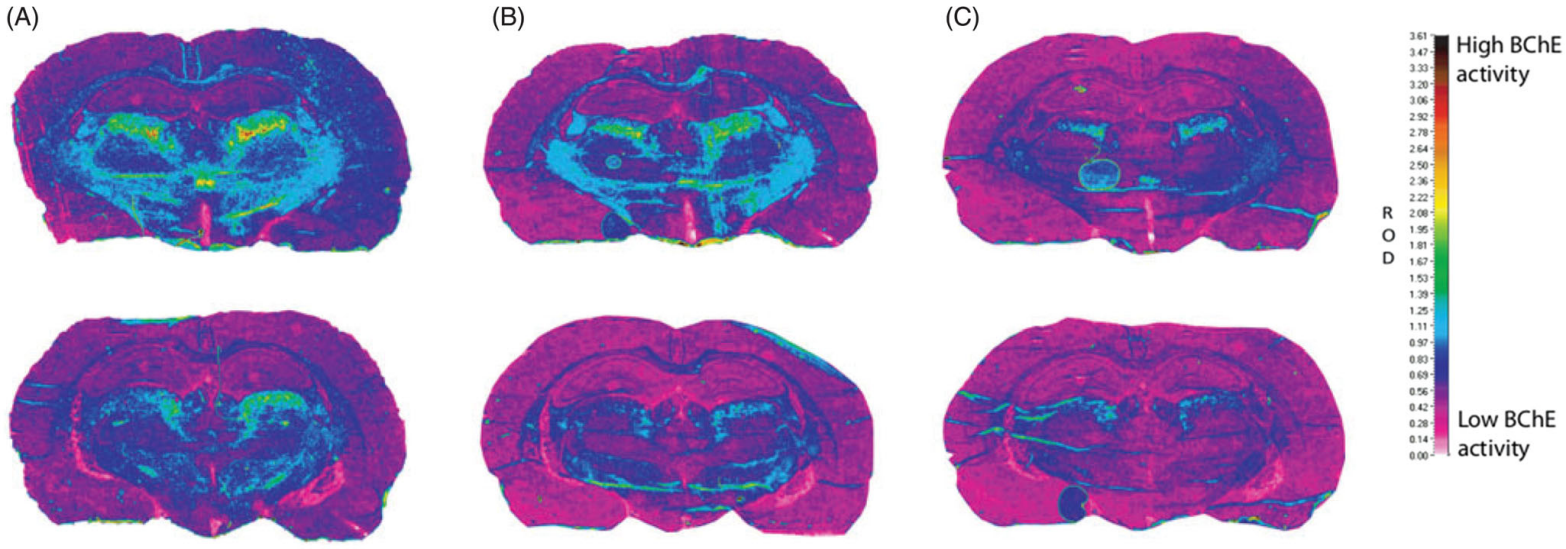
Histochemical staining for BChE activity of coronal cryosections (10 μm) of a rat brain from two regions (top/bottom row) at the level of the thalamus. All sections were processed with 0.1 mM BW-284C51 in Koelle solution, to completely block AChE activity. The sections were incubated in the absence of the probes (A) and with probes **2C** (1 mM; B) and **3B** (1 mM; C). The relative optical density (ROD) pseudocolor scale for staining intensity of BChE activity is on the right.

The inhibition of BChE by these highly specific fluorescent probes was pursued with a view to diagnosis and monitoring of progression of AD, by labelling the brain BChE that accumulates in disease-associated Aβ plaques. Inhibition of BChE in rat brain slices was therefore examined for probes **2C** and **3B**, to evaluate these two probes under more biologically relevant conditions. Sections of rat brain with the highest BChE activity were selected and the modified Koelle − Karnovsky histochemical method was used to detect BChE activity^16^^,^^40–42^. For these probes **2C** and **3B**, 1 mM was needed to achieve substantial inhibition of BChE, as measured by less formation of the dark-brown/black granular BChE reaction product (Figure 8). The parent inhibitors **2** and **3** produced similar effects at 300 µM and 10 µM, respectively, which indicated the lower inhibition constants of the parent inhibitors.

## Conclusions

We report here on the design, synthesis, kinetics and spectroscopic characterisation and binding modes of the two potent BChE-specific fluorescent probes. Design of these fluorescent probes was based on the crystal structures of previously described potent cholinesterase inhibitors in complex with their target, huBChE. Initially, a fluorophore was introduced in place of the naphthalene that binds to the acyl binding pocket of BChE, although this approach went no further. Instead, the scaffold was changed to ones that were already optimised to provide the crucial interactions with the active site gorge residues. The fluorophores were then introduced to be positioned to point outside the aromatic site gorge, towards the bulk. This approach was successful, as it yielded three promising probes, **2C**, **3A**, and **3B**, which show good inhibition of BChE in the low nanomolar range while showing negligible inhibition of AChE. The fluorescence properties of the most promising probes, **2A** and **3A**, show that they both have environment-sensitive emission. Most notably, the emission of probe **2C** is significantly enhanced upon binding to the active site, and thus can be used as a tool to study the activity of BChE. Additionally, inhibition of BChE activity of rat brain slices was investigated with probes **2C** and **3B**, as measured by the modified Koelle − Karnovsky histochemical method. Both probes successfully inhibited formation of the dark-brown/black granular BChE reaction product. Finally, the crystal structures of probes **2C**, **3A**, and **3B** in complex with huBChE were obtained, which provide an excellent starting point for further optimisation of these fluorescent probes for BChE. In particular, the absence of electron density for the fluorophore moieties in these structures indicate that further improvement in inhibition potency and in environment sensitivity of the fluorescence emission can be provided by changes in the linker length and nature, as well as by grafting additional functional groups onto the fluorescent probes. This should provide tighter interactions with the protein scaffold, and thereby better fulfil the requirement for further evaluation as AD-specific fluorescent probes.

## Supplementary Material

Supplemental Material
