# Supplementary material for: Development of potent reversible selective inhibitors of butyrylcholinesterase as fluorescent probes
Source: J Enzyme Inhib Med Chem. 2020 Jan 8;35(1):498–505. doi: 10.1080/14756366.2019.1710502 (PMC6968640; doi:10.1080/14756366.2019.1710502)
Supplement: Supplemental Material [file IENZ_A_1710502_SM7888.pdf]

Supplemental material

Development of Potent Reversible Selective Inhibitors of Butyrylcholinesterase as Fluorescent Probes

Stane Pajk,[a] Damijan Knez,[a] Urban Košak,[a] Maja Zorović,[b] Xavier Brazzolotto,[c] Nicolas Coquelle,[d] Florian Nachon,[c] Jacques-Philippe Colletier,[e] Marko Živin,[b] Jure Stojan,[f] Stanislav Gobec[a]*

[a]*Faculty of Pharmacy, University of Ljubljana, Aškerčeva 7, 1000 Ljubljana, Slovenia;* [b]*Institute of Pathological Physiology, Faculty of Medicine, University of Ljubljana, Vrazov trg 2, 1000 Ljubljana, Slovenia;* [c]*Institut de Recherche Biomédicale des Armées, Département de Toxicologie et Risques Chimiques, 91223, Brétigny sur Orge, France;* [d]*Institut Laue Langvein, 71 Avenue des Martyrs, 38000 Grenoble, France;* [e]*Université Grenoble Alpes, CNRS CEA, IBS, F-38044 Grenoble, France;* [f]*Institute of Biochemistry, Faculty of Medicine, University of Ljubljana, Vrazov trg 2, 1000 Ljubljana, Slovenia*

*correspondence: stanislav.gobec.@ffa.uni-lj.si

1. Synthesis

1.1 General

Chemicals from Sigma-Aldrich, TCI, and Acros were used without further purification. All reactions were performed under argon atmosphere, unless otherwise stated. Analytical TLC was performed on Merck silica gel (60 F_{254}) plates (0.25 mm) and visualized with ultraviolet light (254 or 366 nm). ^{1}H and ^{13}C NMR spectra were recorded on a Bruker AVANCE III 400 MHz NMR spectrometer in $CDCl_3$ and DMSO-d_6 solution, with TMS or residual non-deuterated solvent as the internal standards. Mass spectra were recorded using a VG-Analytical Q-TOF Premier mass spectrometer.

1.2 Synthesis of compond **1A**

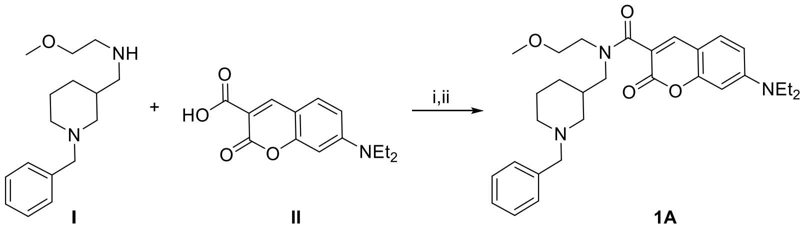

Scheme S1. Reagents and conditions: (i) oxalyl chloride, DMF, DCM, 0 °C to reflux; (ii) Et_3N, 0 °C to rt, 15 h.

Compounds **I**[1] and **II**[2] were synthesized according to the published procedures.

***N*-((1-Benzylpiperidin-3-yl)methyl)-7-(diethylamino)-*N*-(2-methoxyethyl)-2-oxo-2*H*-chromene-3-carboxamide** (**1A**). 7-(Diethylamino)-2-oxo-2*H*-chromene-3-carboxylic acid (**I**) (112 mg, 0.43 mmol, 1.1 equiv) was suspended in CH_2Cl_2 (5 mL) and cooled to 0 °C. The solution was stirred and dimethylformamide (10 µL) was added, followed by dropwise addition of oxalyl chloride (48 µL, 0.56 mmol, 1.3 equiv). The reaction mixture was stirred at 0 °C for 5 minutes, then at reflux conditions for 30 minutes. Solvent was removed under reduced pressure and the residue was dissolved in CH_2Cl_2 (5 mL) and cooled to 0 °C. This solution was added dropwise to the solution of *N*-((1-benzylpiperidin-3-yl)methyl)-2-methoxyethan-1-amine (**II**) (113 mg, 0.39 mmol, 1.0 equiv) and Et_3N (119 µL, 0.86 mmol, 2.0 equiv) in CH_2Cl_2 (5 mL) cooled to 0 °C. The reaction mixture was allowed to reach room temperature and stirred for 15 h. The mixture was transferred to separating funnel, washed with water (20 mL), saturated aqueous $NaHCO_3$ (20 mL), brine (10 mL) and dried over anhydrous Na_2SO_4. Solvent was evaporated to produce the crude product, which was purified by flash column chromatography using DCM/MeOH (from 100:1 to 30:1, v/v) as the eluent to produce 78 mg of compound **1A** as a yellow solid (39% yield).

^{1}H NMR (400 MHz, $CDCl_3$) δ 8.47 (s, 0.03H), 7.54 (s, 0.47H), 7.51 (s, 0.50H), 7.37 – 7.09 (m, 6H), 6.51 (dd, *J* = 8.9, 2.3 Hz, 1H), 6.38 (dd, *J* = 5.3, 2.3 Hz, 1H), 3.68 – 3.47 (m, 3H),

3.47 – 3.24 (m, 9H), 3.23 – 3.06 (m, 3H), 2.95 – 2.50 (m, 2H), 2.26 – 1.87 (m, 2H), 1.84 – 1.34 (m, 4H), 1.22 – 0.92 (m, 7H).

^{13}C NMR (101 MHz, $CDCl_3$) δ 167.13, 166.79, 159.17, 159.00, 156.94, 156.87, 151.33, 151.27, 143.40, 143.22, 137.50, 136.45, 129.72, 129.52, 129.14, 128.24, 128.11, 127.36, 127.02, 117.49, 109.15, 107.59, 107.48, 96.95, 70.46, 70.19, 63.20, 62.94, 58.90, 58.80, 57.27, 57.08, 53.61, 53.53, 53.48, 48.41, 48.03, 45.74, 45.40, 44.83, 35.09, 34.18, 28.14, 27.83, 24.44, 24.21, 12.37, 8.81.

HRMS (ESI): *m/z* calcd for $C_{30}H_{40}N_3O_4$ $[M+H]^+$ 506.3019; found 506.3011.

1.3 Synthesis of compounds **2A** and **2B**

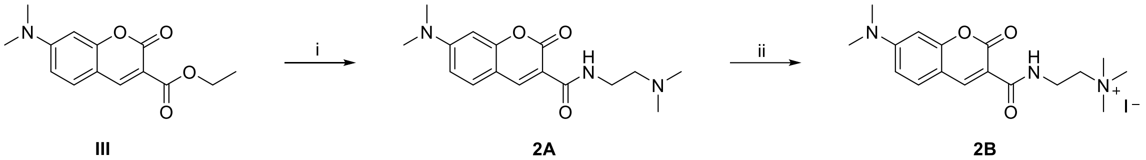

Scheme S2. Reagents and conditions: (i) N^1,N^1-dimethylethane-1,2-diamine, EtOH, reflux, 15 h; (ii) MeI, DCM, rt, 24 h.

Compound **III**[3] was synthesized according to the published procedure.

7-(Dimethylamino)-*N*-(2-(dimethylamino)ethyl)-2-oxo-2*H*-chromene-3-carboxamide (**2A**). Ethyl 7-(dimethylamino)-2-oxo-2*H*-chromene-3-carboxylate (300 mg, 1.15 mmol, 1.0 equiv) and N^1,N^1-dimethylethane-1,2-diamine (286 µL, 2.3 mmol, 2.0 equiv) were suspended in dry ethanol (30 mL) and refluxed for 15 h. Solvent was removed under reduced pressure and the residue was dissolved in DCM (50 mL). The solution was washed with water (5×50 mL), brine (50 mL) and dried over anhydrous Na_2SO_4. The solvent was evaporated and the product was purified by flash column chromatography using DCM/MeOH (from 20:1 to 10/1, v/v) as the eluent to produce 225 mg of compound **2B** as a yellow solid (64% yield).

^{1}H NMR (400 MHz, MeOD) δ 7.54 (d, J = 9.0 Hz, 1H), 6.81 (dd, J = 9.0, 2.4 Hz, 1H), 6.55 (d, J = 2.3 Hz, 1H), 3.56 (t, J = 6.6 Hz, 2H), 2.61 (t, J = 6.6 Hz, 2H).

^{13}C NMR (101 MHz, TFA-d) δ 167.36, 163.93, 156.82, 152.12, 148.66, 135.67, 122.26, 120.32, 120.18, 112.06, 61.12, 49.32, 45.83, 38.21.

HRMS (ESI): *m/z* calcd for $C_{16}H_{22}N_3O_3$ $[M+H]^+$ 304.1656; found 304.1647.

2-(7-(Dimethylamino)-2-oxo-2*H*-chromene-3-carboxamido)-*N*,*N*,*N*-trimethylethan-1-aminium iodide (**2B**)

7-(Dimethylamino)-*N*-(2-(dimethylamino)ethyl)-2-oxo-2*H*-chromene-3-carboxamide (**2A**) (100 mg, 0.33 mmol, 1.0 equiv) was dissolved in DCM (10 mL) followed by addition of MeI (27 µL, 0.43 mmol,1.3 equiv). Product started to precipitate while reaction mixture was stirred at room temperature for 24 h. Product was filtered off and dried to give 95 mg of compound **2C** as a yellow solid (65% yield).

^{1}H NMR (400 MHz, MeOD) δ 7.60 (d, *J* = 9.0 Hz, 1H), 6.87 (dd, *J* = 9.0, 2.5 Hz, 1H), 6.61 (d, *J* = 2.3 Hz, 1H), 3.92 – 3.87 (m, 2H), 3.58 (t, *J* = 6.6 Hz, 2H).

^{13}C NMR (101 MHz, TFA-d) δ 166.03, 163.79, 156.68, 152.31, 148.63, 135.55, 122.33, 120.58, 120.45, 112.37, 66.25, 55.68, 49.09, 36.49.

HRMS (ESI): *m/z* calcd for $C_{17}H_{24}N_3O_3$ $[M]^+$ 318.1812; found 318.1805.

1.4 Synthesis of probe **2C**

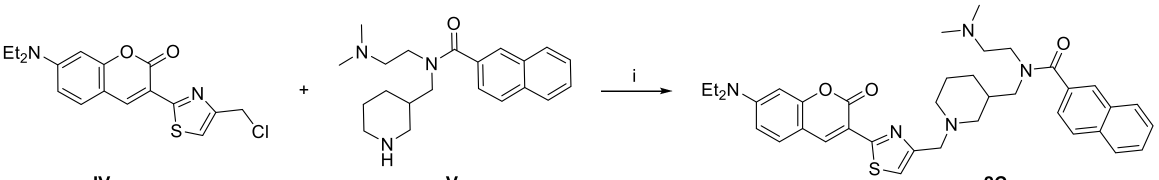

Scheme S3. Reagents and conditions: (i) K_2CO_3, ACN, 60 °C, 48 h.

Compounds **IV**[4] and **V**[5] were synthesized according to the published procedures.

***N*-((1-((2-(7-(Diethylamino)-2-oxo-2*H*-chromen-3-yl)thiazol-4-yl)methyl)piperidin-3-yl)methyl)-*N*-(2-(dimethylamino)ethyl)-2-naphthamide** (**2C**). K_2CO_3 (47 mg, 0.34 mmol, 2.0 equiv), 3-(4-(chloromethyl)thiazol-2-yl)-7-(diethylamino)-2*H*-chromen-2-one (**IV**) (62 mg, 0.18 mmol, 1 equiv), and *N*-(2-(dimethylamino)ethyl)-*N*-(piperidin-3-ylmethyl)-2-naphthamide (**V**) (60 mg, 0.18 mmol, 1 equiv) were suspended in acetonitrile (5 mL) and the reaction mixture was stirred at 60 °C for 48 h. The solvent was removed under reduced pressure and the crude product was purified by flash column chromatography using DCM/MeOH/$NH_{4(aq)}$ (100:5:1, v/v) as the eluent to produce 38 mg of probe **2C** as an orange solid (32% yield).

^{1}H NMR (400 MHz, $CDCl_3$) δ 8.61 (s, 1H), 7.88 – 7.57 (m, 4H), 7.53 – 6.94 (m, 5H), 6.65 – 6.25 (m, 2H), 3.82 – 3.12 (m, 9H), 2.97 (dd, *J* = 47.7, 8.5 Hz, 1H), 2.85 – 2.67 (m, 1H), 2.56 (bs, 1H), 2.37 – 2.17 (m, 4H), 2.14 – 2.04 (bs, 1H), 1.96 – 1.80 (m, 5H), 1.76 – 1.31 (m, 4H), 1.27 – 1.01 (m, 7H).

^{13}C NMR (101 MHz, $CDCl_3$) δ 172.42, 161.07, 160.56, 156.36, 153.15, 151.45, 140.46, 140.18, 134.32, 133.41, 133.35, 132.72, 132.66, 130.44, 129.45, 129.19, 128.31, 127.87, 127.11, 126.88, 126.65, 126.04, 124.54, 124.07, 118.15, 117.67, 112.89, 109.63, 108.58, 96.86, 63.58, 58.91, 57.72, 57.47, 56.79, 54.81, 53.54, 50.65, 48.16, 47.37, 45.78, 45.60, 45.02, 43.04, 35.30, 28.48, 25.00, 12.54.

HRMS (ESI): *m/z* calcd for $C_{38}H_{45}N_5O_3S$ $[M+H]^+$ 652.3321; found 652.3320.

1.5 Synthesis of probe **3A**

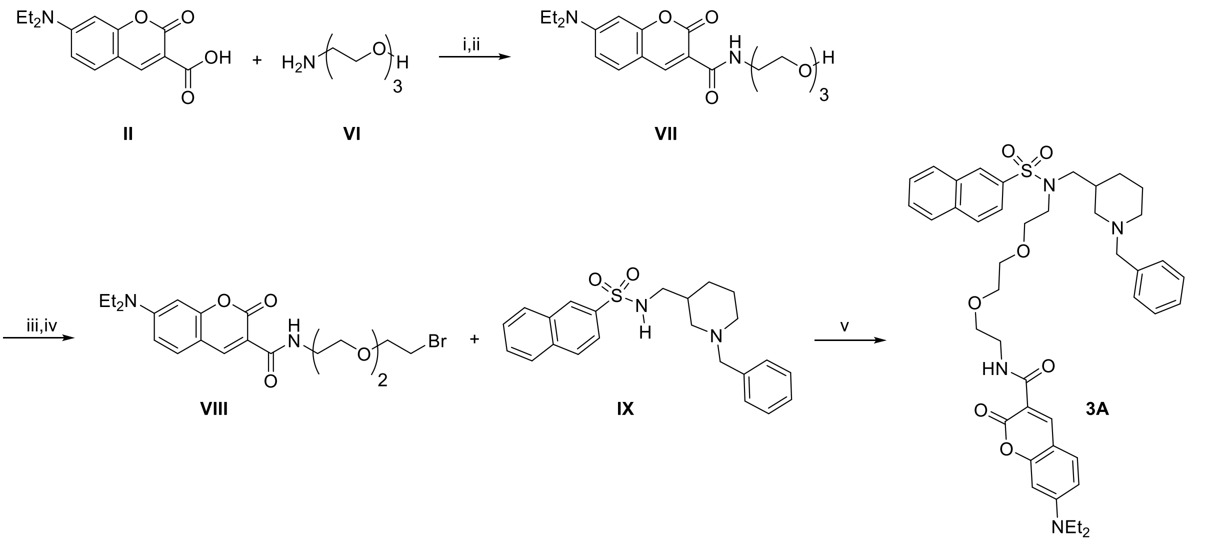

Scheme S4. Reagents and conditions: (i) oxalyl chloride, DMF, DCM, 0 °C to reflux; (ii) Et_3N, 0 °C to rt, 15 h; (iii) mesyl chloride, DCM, 0 °C to rt, 1 h; (iv) LiBr, acetone, 60 °C, 15 h; (v) Cs_2CO_3, ACN, 60 °C, 24 h.

Compounds **VI**[6] and **IX**[1] were synthesized according to the published procedures.

7-(Diethylamino)-*N*-(2-(2-(2-hydroxyethoxy)ethoxy)ethyl)-2-oxo-2*H*-chromene-3-carboxamide (VII).

7-(Diethylamino)-2-oxo-2*H*-chromene-3-carboxylic acid (**II**) (515 mg, 1.97 mmol, 1.0 equiv) was suspended in CH_2Cl_2 (25 mL) and cooled to 0 °C. The solution was stirred and dimethylformamide (50 µL) was added, followed by dropwise addition of oxalyl chloride (334 µL, 3.94 mmol, 2.0 equiv). The reaction mixture was stirred at 0 °C for 5 minutes, then at reflux conditions for 30 minutes. Solvent was removed under reduced pressure and the residue was dissolved in CH_2Cl_2 (10 mL) and cooled to 0 °C. This solution was added dropwise to the solution of 2-(2-(2-aminoethoxy)ethoxy)ethan-1-ol (**VI**) (440 mg, 2.95 mmol, 1.5 equiv) and Et_3N (327 µL, 2.24 mmol, 1.2 eqiuv) in CH_2Cl_2 (25 mL) cooled to 0 °C. The reaction mixture was allowed to reach room temperature and stirred for 2 h. The mixture was

transferred to separating funnel, washed with 1 M HCl (25 mL), brine (15 mL) and dried over anhydrous Na_2SO_4. Solvent was evaporated to produce the crude product, which was purified by flash column chromatography using DCM/MeOH (from 50:1 to 10:1, v/v) as the eluent to produce 422 mg of compound **VII** as a yellow oil (54% yield).

^{1}H NMR (400 MHz, $CDCl_3$) δ 9.15 – 9.05 (m, 1H), 8.70 (s, 1H), 7.43 (d, *J* = 9.0 Hz, 1H), 6.65 (dd, *J* = 9.0, 2.4 Hz, 1H), 6.50 (d, *J* = 2.4 Hz, 1H), 3.80 – 3.75 (m, 2H), 3.75 – 3.60 (m, 10H), 3.45 (q, *J* = 7.1 Hz, 4H), 1.24 (t, *J* = 7.1 Hz, 6H).

HRMS (ESI): *m/z* calcd for $C_{20}H_{29}N_2O_6$ $[M+H]^+$ 393.2026; found 393.2020.

_N_-(2-(2-(2-Bromoethoxy)ethoxy)ethyl)-7-(diethylamino)-2-oxo-2_H_-chromene-3-carboxamide (VIII).

7-(Diethylamino)-*N*-(2-(2-(2-hydroxyethoxy)ethoxy)ethyl)-2-oxo-2*H*-chromene-3-carboxamide (**IX**) (403 mg, 1.03 mmol, 1 eqiuv) was dissolved CH_2Cl_2 (15 mL) and cooled to 0 °C. The solution was stirred and Et_3N (185 µL, 134 mmol, 1.3 equiv) was added, followed by dropwise addition of mesyl chloride (88 µL, 1.13 mmol, 1.1 eqiuv). The reaction mixture was allowed to reach room temperature and stirred for 1 h. The mixture was transferred to separating funnel, washed with 1 M HCl (25 mL), brine (20 mL) and dried over anhydrous Na_2SO_4. Solvent was evaporated and the residue was dissolved in acetone (20 mL). LiBr (1.77 g, 20.6 mmol, 20 equiv) was added portionwise to the stirred solution. The reaction mixture was heated to 60 °C and stirred for 15 h. Solvent was evaporated, and the residues suspended in EtOAc (30 mL). The mixture was transferred to separating funnel, washed with water (30 mL), brine (20 mL) and dried over anhydrous Na_2SO_4. Solvent was evaporated to produce the crude product, which was purified by flash column chromatography using EtOAc/Hex (from 1:2 to 3:1, v/v) as the eluent to produce 194 mg of compound **VIII** as a yellow oil (41% yield).

^{1}H NMR (400 MHz, $CDCl_3$) δ 9.03 (t, *J* = 4.7 Hz, 1H), 8.69 (s, 1H), 7.42 (d, *J* = 9.0 Hz, 1H), 6.64 (dd, *J* = 9.0, 2.5 Hz, 1H), 6.49 (d, *J* = 2.3 Hz, 1H), 3.85 (t, *J* = 6.3 Hz, 1H), 3.75 – 3.62 (m, 4H), 3.50 (t, *J* = 6.3 Hz, 1H), 3.45 (q, *J* = 7.1 Hz, 2H), 1.24 (t, *J* = 7.1 Hz, 3H).

HRMS (ESI): *m/z* calcd for $C_{20}H_{28}N_2O_5Br$ $[M+H]^+$ 455.1182; found 455.1185.

_N_-(2-(2-(2-(_N_-((1-Benzylpiperidin-3-yl)methyl)naphthalene-2-sulfonamido)ethoxy)ethoxy)ethyl)-7-(diethylamino)-2-oxo-2_H_-chromene-3-carboxamide (3A).

N-((1-Benzylpiperidin-3-yl)methyl)naphthalene-2-sulfonamide (**IX**) (48 mg, 0.105 mmol, 1 equiv), Cs_2CO_3 (68 mg, 0.21 mmol, 2 equiv) and *N*-(2-(2-(2-bromoethoxy)ethoxy)ethyl)-7-(diethylamino)-2-oxo-2*H*-chromene-3-carboxamide (**VIII**) (42 mg, 0.105 mmol, 1 equiv) were dissolved in ACN (3 mL) and stirred at 60 °C for 24 h. The solvent was evaporated and

the product was purified by flash column chromatography using DCM/MeOH (from 60:1 to 30:1, v/v) as the eluent to produce 55 mg of probe **3A** as a yellow oil (68% yield).

^{1}H NMR (400 MHz, $CDCl_3$) δ 8.97 (t, *J* = 5.2 Hz, 1H), 8.66 (d, *J* = 0.4 Hz 1H), 8.41 – 8.35 (m, 1H), 8.01 – 7.85 (m, 3H), 7.77 (ddd, *J* = 8.7, 3.7, 1.9 Hz, 1H), 7.65 – 7.54 (m, 2H), 7.39 (d, *J* = 9.0 Hz, 1H), 7.31 – 7.18 (m, 5H), 6.61 (dd, *J* = 9.0, 2.3 Hz, 1H), 6.45 (d, *J* = 2.3 Hz, 1H), 3.64 – 3.51 (m, 6H), 3.50 – 3.46 (m, 4H), 3.42 (q, *J* = 7.1 Hz, 4H), 3.36 (td, *J* = 6.3, 2.2 Hz, 2H), 3.19 – 3.08 (m, 2H), 2.91 – 2.78 (m, 2H), 2.75 – 2.51 (m, 2H), 2.16 – 1.91 (m, 1H), 1.88 – 1.37 (m, 5H), 1.21 (t, *J* = 7.1 Hz, 6H), 1.10 – 0.92 (m, 1H).

^{13}C NMR (101 MHz, $CDCl_3$) δ 163.32, 162.60, 157.70, 152.56, 148.09, 138.12, 137.89, 136.80, 136.70, 134.78, 134.73, 132.23, 132.19, 131.19, 129.51, 129.29, 129.19, 128.76, 128.67, 128.48, 128.42, 128.33, 128.23, 127.96, 127.91, 127.57, 127.51, 127.19, 127.07, 122.72, 122.40, 110.30, 109.95, 108.38, 96.58, 70.44, 70.31, 69.86, 69.80, 63.39, 57.76, 57.26, 53.93, 53.22, 48.21, 47.24, 45.12, 39.44, 34.77, 28.22, 24.59, 24.16, 12.49.

HRMS (ESI): *m/z* calcd for $C_{43}H_{53}N_4O_7S$ $[M+H]^+$ 769.3635; found 769.3630.

1.6 Synthesis of probe **3B**

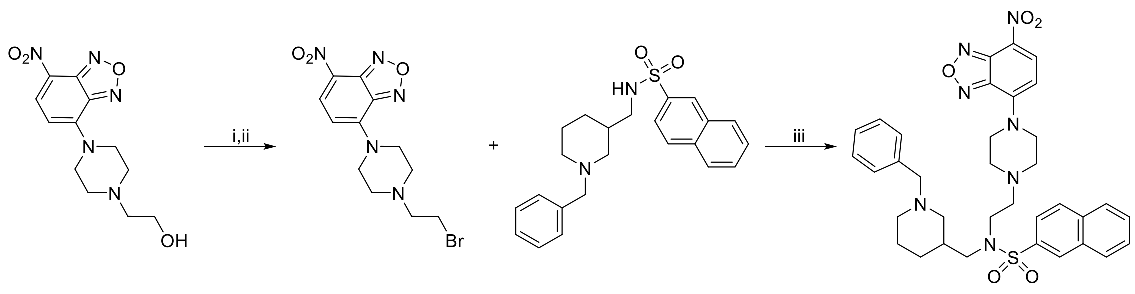

X XI IX 3B

Scheme S5. Reagents and conditions: (i) mesyl chloride, DCM, 0 °C to rt, 30 min; (ii) LiBr, acetone, 50 °C, 1 h; (iii) Cs_2CO_3, ACN, 60 °C, 24 h.

Compound **X**[7] was synthesized according to the published procedure.

4-(4-(2-Bromoethyl)piperazin-1-yl)-7-nitrobenzo[c][1,2,5]oxadiazole (XI).

2-(4-(7-Nitrobenzo[c][1,2,5]oxadiazol-4-yl)piperazin-1-yl)ethan-1-ol (**X**) (560 mg, 1.91 mmol, 1.0 equiv) was dissolved CH_2Cl_2 (5 mL) and cooled to 0 °C. The solution was stirred and Et_3N (528 µL, 3.82 mmol, 2.0 equiv) was added, followed by dropwise addition of mesyl chloride (155 µL, 2.0 mmol, 1.05 equiv). The reaction mixture was allowed to reach room temperature and stirred for 30 min. DCM (25 mL) was added to the reaction mixture, and the solution was transferred to separating funnel, washed with water (20 mL), saturated aqueous

$NaHCO_3$ solution (20 mL), brine (15 mL) and dried over anhydrous Na_2SO_4. Solvent was evaporated and the residue was dissolved in acetone (10 mL). The solution was stirred, and LiBr (1.66 g, 19.1 mmol, 10 equiv) was added. The reaction mixture was heated to 50 °C and stirred for 1 h. Solvent was evaporated and the residue was suspended in EtOAc (20 mL). The mixture was transferred to separating funnel, washed with water (20 mL), brine (20 mL) and dried over anhydrous Na_2SO_4. Solvent was evaporated to produce 467 mg of crude product (69%), which was used immediately in the next reaction step without further purification.

^{1}H NMR (400 MHz, DMSO-d_6) δ 8.47 (d, J = 9.2 Hz, 1H), 6.69 (d, J = 9.2 Hz, 1H), 4.13 (apps, 4H), 3.63 (t, J = 6.9 Hz, 2H), 2.81 (apps, 2H), 2.74 (apps, 4H).

***N*-((1-Benzylpiperidin-3-yl)methyl)-*N*-(2-(4-(7-nitrobenzo[c][1,2,5]oxadiazol-4-yl)piperazin-1-yl)ethyl)naphthalene-2-sulfonamide** (**3B**).

N-((1-Benzylpiperidin-3-yl)methyl)naphthalene-2-sulfonamide (**IX**) (180 mg, 0.45 mmol, 1.0 equiv), Cs_2CO_3 (222 mg, 0.68 mmol, 1.5 equiv) and 4-(4-(2-Bromoethyl)piperazin-1-yl)-7-nitrobenzo[c][1,2,5]oxadiazole (**XI**) (162 mg, 0.45 mmol, 1.0 equiv) were dissolved in ACN (5 mL) and stirred at 60 °C for 24 h. The solvent was evaporated under reduced pressure and the residue suspended in EtOAc (30 mL). The mixture was transferred to separating funnel, washed with water (20 mL), saturated aqueous $NaHCO_3$ solution (20 mL), brine (20 mL) and dried over anhydrous Na_2SO_4. The solvent was evaporated and the product was purified by flash column chromatography using DCM/MeOH/$NH_{4(aq)}$ (from 100:2:1 to 100:10:1, v/v) as the eluent to produce 47 mg of probe **3B** as a yellow solid (15% yield).

^{1}H NMR (400 MHz, $CDCl_3$) δ 8.38 – 8.36 (m, 1H), 8.34 (d, J = 9.1 Hz, 1H), 8.00 – 7.87 (m, 3H), 7.76 (dd, J = 8.7, 1.9 Hz, 1H), 7.67 – 7.58 (m, 2H), 7.30 – 7.13 (m, 5H), 6.22 (d, J = 9.1 Hz, 1H), 4.06 – 3.92 (m, 4H), 3.44 (d, J = 2.8 Hz, 2H), 3.37 – 3.19 (m, 2H), 3.11 (d, J = 7.6 Hz, 2H), 2.90 – 2.42 (m, 8H), 2.05 – 1.85 (m, 2H), 1.80 – 1.61 (m, 3H), 1.60 – 1.44 (m, 1H), 1.08 – 0.89 (m, 1H).

^{13}C NMR (101 MHz, $CDCl_3$) δ 145.13, 144.75, 138.27, 136.32, 135.25, 134.71, 132.16, 129.45, 129.17, 129.14, 128.87, 128.46, 128.19, 127.95, 127.71, 127.01, 123.21, 122.50, 102.62, 63.43, 57.74, 56.81, 54.09, 53.26, 52.76, 49.31, 46.26, 35.13, 28.43, 24.72.

HRMS (ESI): *m/z* calcd for $C_{35}H_{40}N_7O_5S$ $[M+H]^+$ 670.2812; found 670.2821.

1.7 Synthesis of compounds **3C** and **3D**

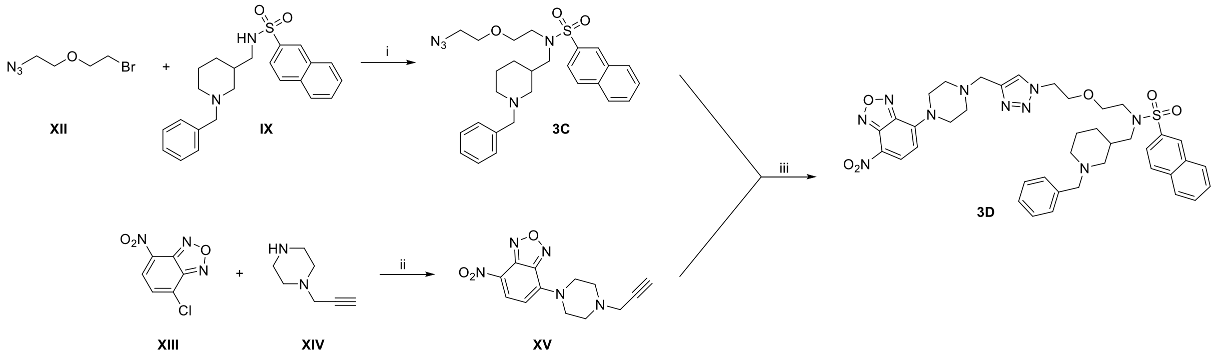

Scheme S6. Reagents and conditions: (i) Cs_2CO_3, ACN, 60 °C, 3 d; (ii) K_2CO_3, THF, 0 °C to rt, 24 h; (iii) CuBr, *N*-methylmorpholine, EtOAc, 40 °C, 24 h.

Compounds **XII**[8] and **XIV**[9] were synthesized according to the published procedures.

_N_-(2-(2-Azidoethoxy)ethyl)-N-((1-benzylpiperidin-3-yl)methyl)naphthalene-2-sulfonamide (**3C**).

N-((1-Benzylpiperidin-3-yl)methyl)naphthalene-2-sulfonamide (**IX**) (100 mg, 0.25 mmol, 1.0 equiv), 1-azido-2-(2-bromoethoxy)ethane (**XII**) (74 mg, 0.38 mg, 1.5 equiv) and Cs_2CO_3 (165.5 mg, 0.51 mmol, 2.0 equiv) were suspended in ACN (5 mL), and stirred at 60 °C for 3 days. Solvents were removed under reduced pressure and the crude product was purified by flash column chromatography using DCM/MeOH (from 9:1 to 9:2, v/v) as the eluent to produce 71 mg of azide **3C** as a yellow viscous oil (56% yield).

^{1}H NMR (400 MHz, $CDCl_3$) δ 8.41 – 8.35 (m, 1H), 8.01 – 7.86 (m, 3H), 7.76 (dd, *J* = 8.6, 1.9 Hz, 1H), 7.68 – 7.58 (m, 2H), 7.43 – 7.18 (m, 5H), 3.58 (t, *J* = 6.2 Hz, 2H), 3.51 – 3.46 (m, 4H), 3.37 – 3.20 (m, 4H), 3.13 (d, *J* = 7.5 Hz, 2H), 2.77 (dd, *J* = 32.4, 9.2 Hz, 2H), 2.05 – 1.90 (d, 2H), 1.85 – 1.40 (m, 4H), 1.08 – 0.92 (m, 1H).

HRMS (ESI): *m/z* calcd for $C_{27}H_{34}N_5O_3S$ $[M+H]^+$ 508.2377; found 508.2384.

4-Nitro-7-(4-(prop-2-yn-1-yl)piperazin-1-yl)benzo[*c*][1,2,5]oxadiazole (XV).

Solution of NBDCl (**XIII**) (50 mg, 0.25 mmol, 1.0 equiv) in THF (5 mL) was added dropwise to the mixture of 1-(prop-2-yn-1-yl)piperazine (**XIV**) (48.5 mg, 0.30 mmol, 1.2 equiv) and K_2CO_3 (172 mg, 1.25 mmol, 1.5 equiv) in THF (5 mL). During the addition, the reaction mixture was vigorously stirred and cooled on an ice bath. Following the addition, the temperature of the reaction mixture was allowed to reach room temperature and stirring was continued for 24 h. The solvent was evaporated under reduced pressure and the residue suspended in DCM (30 mL). The mixture was transferred to separating funnel, washed with

water (20 mL), saturated aqueous $NaHCO_3$ solution (20 mL), brine (20 mL) and dried over anhydrous Na_2SO_4. The solvent was evaporated under reduced pressure to produce 51 mg of the desired product **XV** as a red powder (71% yield).

^{1}H NMR (400 MHz, DMSO-d$_6$) δ 8.48 (d, *J* = 9.2 Hz, 1H), 6.69 (d, *J* = 9.2 Hz, 1H), 4.19 – 4.12 (m, 4H), 3.40 (d, *J* = 2.4 Hz, 2H), 3.21 (t, *J* = 2.4 Hz, 1H), 2.72 – 2.68 (m, 4H).

MS (ESI): *m/z* calcd for $C_{13}H_{14}N_5O_3$ $[M+H]^+$ 288.11; found 288.21.

_N_-((1-Benzylpiperidin-3-yl)methyl)-_N_-(2-(2-(4-((4-(7-nitrobenzo[_c_][1,2,5]oxadiazol-4-yl)piperazin-1-yl)methyl)-1_H_-1,2,3-triazol-1-yl)ethoxy)ethyl)naphthalene-2-sulfonamide (3D)

4-Nitro-7-(4-(prop-2-yn-1-yl)piperazin-1-yl)benzo[*c*][1,2,5]oxadiazole (**XV**) (8.0 mg, 0.025 mmol, 1.0 equiv) and azide **3C** (12.7 mg, 0.025 mmol. 1.0 equiv) were dissolved in EtOAc (20 mL). *N*-Methylmorpholine (5.5 µl, 0.05 mmol, 2.0 equiv) and catalytic amount of CuBr (~ 1 mg) were added to the stirred mixture at room temperature. The reaction mixture was than stirred at 40 °C for 1 day. The solvents were removed under reduced pressure and the crude product was purified by flash column chromatography using DCM/MeOH (from 40:1 to 10:1, v/v) as the eluent to produce 17 mg of compound **3D** as an orange solid (86% yield).

^{1}H NMR (400 MHz, $CDCl_3$) δ 8.35 (d, *J* = 9.0 Hz, 1H), 8.33 (d, *J* = 1.5 Hz, 1H), 8.00 – 7.86 (m, 3H), 7.79 (s, 1H), 7.72 – 7.58 (m, 3H), 7.36 – 7.20 (m, 5H), 6.23 (d, *J* = 9.0 Hz, 1H), 4.49 (t, *J* = 5.0 Hz, 2H), 4.18 – 4.00 (m, 4H), 3.79 (s, 2H), 3.79 (t, *J* = 5.0 Hz, 2H), 3.86 – 3.44 (m, 4H), 3.42 – 3.18 (m, 2H), 3.14 – 2.86 (m, 4H), 2.81 – 2.72 (m, 4H), 2.15 – 1.15 (m, 4H), 1.40 – 1.15 (m, 2H), 1.05 – 0.70 (m, 1H).

HRMS (ESI): *m/z* calcd for $C_{40}H_{47}N_{10}O_6S$ $[M+H]^+$ 795.3395; found 795.3398.

2. Crystal structure refinement statistics

Table S1. Data collection and refinement statistics calculated using Phenix (Adams *et al* Acta Cryst. D 2010;66:213-221). R-work=Σ |Fo - |Fc|| / Σ |Fo|, Fo and Fc are observed and calculated structure factors, R-free set uses about 5% randomly chosen reflections. Statistics for the highest-resolution shell are shown in parentheses.

	**hBChE 2C**	**hBChE 3A**	**hBChE 3B**
Data collection			
**X-ray source**	ESRF ID23-1	ESRF ID23-2	ESRF ID23-1
**Wavelength**	0.9724	0.8726	0.9724
**Resolution range**	47.89 - 2.5 (2.589 - 2.5)	44.93 - 2.75 (2.848 - 2.750)	55.15 - 2.474 (2.562 - 2.474)
**Space group**	I 4 2 2	P 21 21 21	I 4 2 2
**Unit cell**	154.93 154.93 128.03 90 90 90	76.29 80.2 231.35 90 90 90	154.22 154.22 127.85 90 90 90
**Total reflections**	176432 (18342)	195803 (20184)	200463 (22509)
**Unique reflections**	27001 (2689)	37475 (3702)	26287 (2723)
**Multiplicity**	6.5 (6.8)	5.2 (5.5)	7.6 (8.3)
**Completeness (%)**	98.95 (99.67)	99.10 (99.87)	94.63 (99.96)
**Mean I/sigma(I)**	14.58 (2.55)	12.71 (2.27)	19.30 (2.24)
**Wilson B-factor**	56.69	58.52	60.11
**R-merge**	0.07755 (0.6376)	0.09612 (0.6969)	0.07009 (0.9361)
**R-meas**	0.08423 (0.6882)	0.107 (0.7712)	0.07524 (0.9993)
**R-pim**	0.03107 (0.2458)	0.04593 (0.3244)	0.02689 (0.3458)
**CC1/2**	0.998 (0.823)	0.997 (0.746)	0.999 (0.758)
**CC***	1 (0.95)	0.999 (0.924)	1 (0.929)
Refinement statistics			
**Reflections used in refinement**	26943 (2689)	37465 (3702)	26279 (2725)
**Reflections used for R-free**	1078 (107)	1122 (116)	1296 (129)
**R-work**	0.1922 (0.2379)	0.1811 (0.2546)	0.1823 (0.2711)
**R-free**	0.2572 (0.3616)	0.2375 (0.3398)	0.2275 (0.3606)
**CC(work)**	0.960 (0.880)	0.941 (0.832)	0.958 (0.841)
**CC(free)**	0.916 (0.724)	0.907 (0.696)	0.945 (0.715)
**Number of non-hydrogen atoms**	4588	9029	4521
**macromolecules**	8385	4216	4222
**ligands**	469	214	194
**solvent**	175	91	172
**Protein residues**	537	1053	537
**RMS(bonds)**	0.009	0.006	0.009
**RMS(angles)**	1.05	1.85	1.04
**Ramachandran favored (%)**	95.04	95.33	94.85
**Ramachandran allowed (%)**	4.96	4.39	4.77
**Ramachandran outliers (%)**	0.00	0.29	0.38
**Rotamer outliers (%)**	0.00	1.11	0.00
**Clashscore**	9.23	4.99	9.32
**Average B-factor**	62.11	53.55	66.64
**macromolecules**	51.13	65.01	60.62
**ligands**	99.87	100.27	96.15
**solvent**	45.13	63.40	60.39

3. Rat Brain Slices Experiments

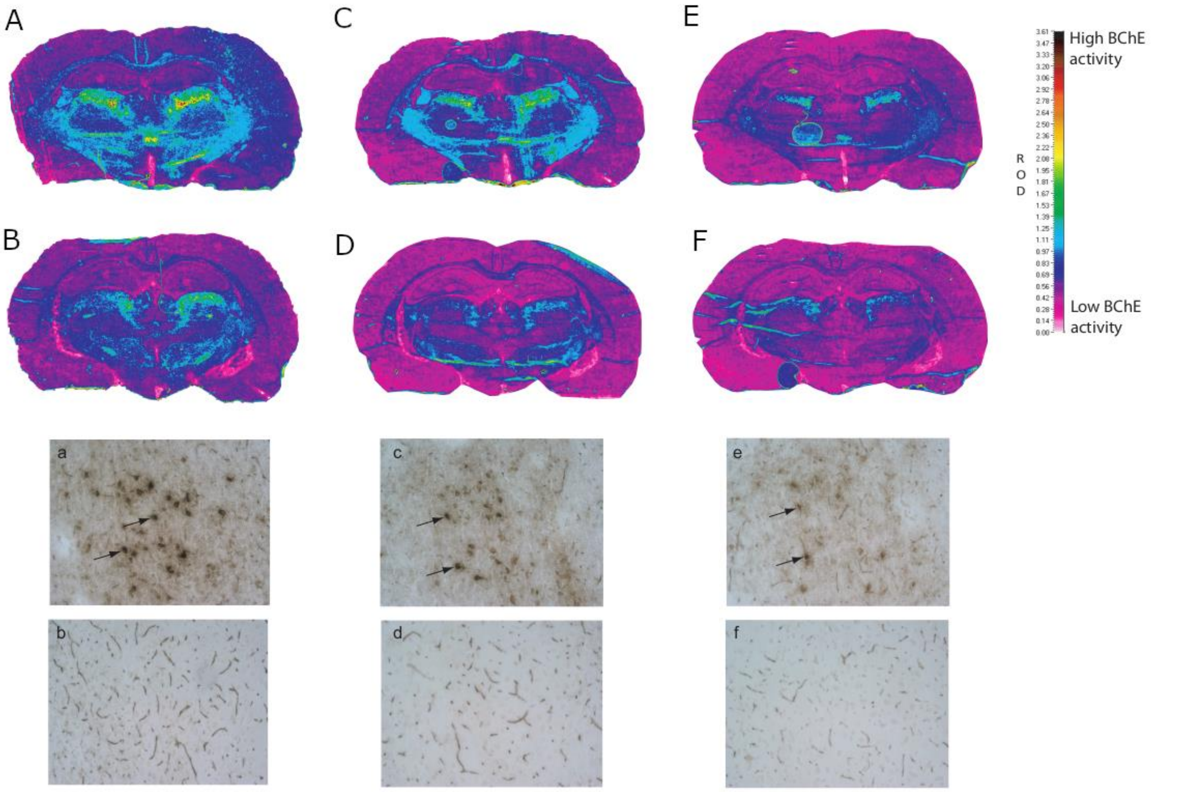

Figure S1. BChE activity histochemical staining of coronal cryosections (10 μm) of a rat brain from two regions at the level of the thalamus. All sections were processed with 0.1 mM BW-284C51 in Koelle solution to completely block the AChE activity (A, B). For the inhibition experiment, the sections were incubated with compounds **2C** (1 mM, C, D) and **3B** (1 mM, E, F), respectively. The relative optical density (ROD) pseudocolor scale for the staining intensity of BChE activity is shown on the right. The photomicrographs show BChE-rich neurons in the laterodorsal thalamic nucleus (arrows) and the capillaries in the cerebral cortex in the absence (a, b) and the presence of compounds **2C** (c, d) and **3B** (e, f). Magnification of the photomicrographs is 20×.

4. Titration of hBChE with probe **2C**

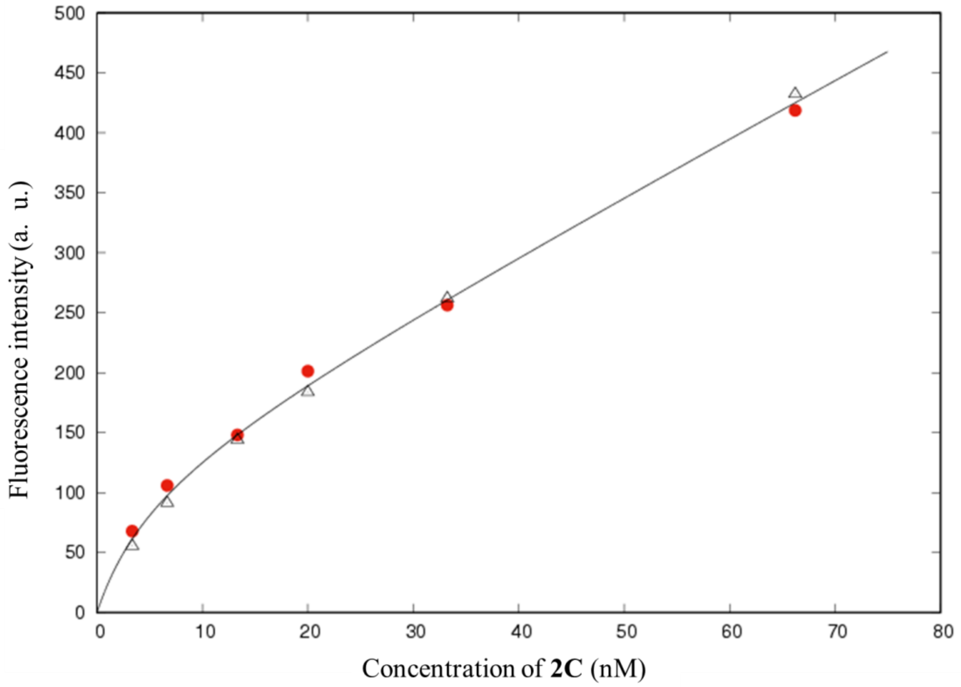

Figure S2. The fluorescence titration data, in duplicate, were simultaneously fitted to the sum of hyperbolic and linear equation. The determined binding constant for probe **2C** was 5.5 +/- 1.6 nM, remarkably similar to the inhibition constant for the binding to the free enzyme (3.3 nM, see Table 1 in the main text).

5. References

1. Košak U, Brus B, Knez D, et al. Development of an *in-vivo* active reversible butyrylcholinesterase inhibitor. Sci Rep. 2016;6:39495.
2. Yao H, Wei G, Liu Y, et al. Synthesis, Biological Evaluation of Fluorescent 23-Hydroxybetulinic Acid Probes, and Their Cellular Localization Studies. ACS Med Chem Lett. 2018;9(10):1030-1034.
3. Hori Y, Norinobu T, Sato M, et al. Development of Fluorogenic Probes for Quick No-Wash Live-Cell Imaging of Intracellular Proteins. J Am Chem Soc. 2013;135(33):12360-12365.
4. Nishizono N, Oda K, Kato Y, et al. Synthesis of Fluorescent Derivatization Reagents: Reaction of Isatin with 3-Aryl-7-diethylaminocoumarins and Their Fluorescent Properties. Heterocycles. 2004;63.
5. Knez D, Coquelle N, Pišlar A, et al. Multi-target-directed ligands for treating Alzheimer's disease: Butyrylcholinesterase inhibitors displaying antioxidant and neuroprotective activities. Eur J Med Chem. 2018;156:598-617.
6. Puchkov PA, Kartashova IA, Shmendel EV, et al. Spacer structure and hydrophobicity influences transfection activity of novel polycationic gemini amphiphiles. Bioorg Med Chem Lett. 2017;27(15):3284-3288.
7. Schininà B, Martorana A, Colabufo NA, et al. 4-Nitro-2,1,3-benzoxadiazole derivatives as potential fluorescent sigma receptor probes. RSC Adv. 2015;5(58):47108-47116.
8. Yu X, Eymur S, Singh V, et al. Flavin as a photo-active acceptor for efficient energy and charge transfer in a model donor–acceptor system. Phys Chem Chem Phys. 2012;14(19):6749-6754.
9. Zheng S, Lingyue G, Ong MJH, et al. Divergent synthesis of 5′,7′-difluorinated dihydroxanthene-hemicyanine fused near-infrared fluorophores. Org Biomol Chem. 2019;17(17):4291-4300.
